# Patching and Suppression in Amblyopia: One Mechanism or Two?

**DOI:** 10.3389/fnins.2019.01364

**Published:** 2020-01-15

**Authors:** Yiya Chen, Zhifen He, Yu Mao, Hao Chen, Jiawei Zhou, Robert F. Hess

**Affiliations:** ^1^School of Ophthalmology and Optometry and Eye Hospital, State Key Laboratory of Ophthalmology, Optometry and Vision Science, Wenzhou Medical University, Wenzhou, China; ^2^McGill Vision Research, Department of Ophthalmology and Visual Sciences, McGill University, Montreal, QC, Canada

**Keywords:** patching, interocular suppression, amblyopia, binocular therapy, visual acuity

## Abstract

**Purpose:**

To determine if benefits from occlusion therapy are due to decreased suppression from the fellow eye in children with amblyopia.

**Methods:**

Ten newly diagnosed amblyopes (7.2 ± 1.4 years old), two with strabismus and eight with anisometropia, participated. Patients were first given a 2-month period of refractive adaptation, followed by occlusion therapy (i.e., patching their fellow eye with an opaque patch for 4 h/day). Visual acuity of the amblyopic eye and interocular suppression were measured before and after 0.5, 1, 2, 4, and 6 months of occlusion therapy. We quantified interocular suppression with a binocular phase combination task.

**Results:**

Visual acuity (in logMAR) improved from 0.50 ± 0.22 (mean ± SD) to 0.33 ± 0.20 for patients who finished a short-term (2 months) occlusion (A1–A10), from 0.53 ± 0.20 to 0.32 ± 0.22 for patients who finished a medium-term (4 months) occlusion (A1–A9), and from 0.48 ± 0.19 to 0.22 ± 0.10 for patients who finished a long-term (6 months) occlusion (A1–A8). Although their visual acuity significantly improved, their degree of suppression, which was abnormal in all cases, did not change consistently. This was true in all durations of occlusion therapy.

**Conclusion:**

Reduced suppression from the fixing eye might not be result from occlusion therapy.

## Introduction

For the past 250 years, occluding the fellow eye has been the standard therapy for amblyopia (de Buffon, 2005). Occlusion therapy “forces” the amblyopic eye to work. Emerging evidence suggests that 120–240 h of occlusion results in a 1-line improvement in visual acuity across all age groups when occlusion is effective (Stewart et al., 2007).

Recently, a new approach has been suggested. It involves re-establishing binocular vision as the first step by using binocularly based training therapy, which relies on a theory that amblyopia and abnormal interocular suppression are causally linked (Baker et al., 2008; Li et al., 2011; Birch, 2013; Ding et al., 2013; Zhou et al., 2013b, c, 2018). It improves not only binocular vision (Li et al., 2013; Hess and Thompson, 2015; Webber et al., 2016; Kelly et al., 2018) but also monocular acuity (Hess et al., 2010b, 2011; To et al., 2011; Li et al., 2013, 2014; Mansouri et al., 2014; Birch et al., 2015). Despite ongoing debate concerning which therapeutic approach is more effective (Holmes et al., 2016; Kelly et al., 2016; Gao et al., 2018; Manh et al., 2018; Pineles et al., 2019), one should bear in mind the possibility that the visual improvements from both approaches might involve the same mechanisms in the brain. If both approaches involve similar neural mechanisms, convenience and efficacy might determine which one to use. Otherwise, they could be used complementarily or specifically according to each patient’s need. At first glance, both occlusion and binocular therapy might work to reduce suppression from the fellow eye which normally prevents the amblyopic eye from improving under normal binocular viewing conditions. Binocular therapy reduces suppression from the fellow eye under binocular viewing by reducing the amount of visual input to the fixing (suppressing) eye. Conversely, occlusion therapy might achieve a similar effect by entirely occluding the fixing (suppressing) eye. However, these two therapeutic protocols could involve disparate neural mechanisms as exemplified by their numerous differences. For example, they differ in the following ways: binocular outcomes [occlusion – poor (Birch, 2013); binocular therapy – good (Hess et al., 2011; Knox et al., 2012; Li et al., 2013; Kelly et al., 2018)], age dependence [occlusion – only children (Epelbaum et al., 1993); binocular therapy – children and adults (Mansouri et al., 2014)], duration dependence [occlusion – 120–240 h (Stewart et al., 2007); binocular therapy – 20–40 h (Hess et al., 2010b; Mansouri et al., 2014)], and post-therapeutic remission [occlusion – 24–27% (Holmes et al., 2004; Bhola et al., 2006); binocular therapy – no remission (Li et al., 2013, 2015; Birch et al., 2015)].

To assess whether occlusion therapy decreases interocular suppression in a similar fashion to binocular therapy (Hess et al., 2010a, b, 2011; Li et al., 2013; Spiegel et al., 2013), we measured interocular suppression at various time points (up to 6 months) in newly diagnosed patients. The patients had been prescribed 4 h per day of occlusion therapy after a 2-month period of refractive adaptation (Wang J. et al., 2018). We used a binocular phase combination task, a standard laboratory method (Ding and Sperling, 2006) to study binocular balance, e.g., amblyopia (Huang et al., 2009; Ding et al., 2013; Zhou et al., 2013b; Kwon et al., 2014) and strabismus (Ding et al., 2013; Feng et al., 2015; Zhou et al., 2017c), and binocular visual plasticity (Zhou et al., 2013a, 2014b, 2017a,b; Min et al., 2019; Sheynin et al., 2019), to quantify the degree of interocular suppression before and during the occlusion therapy. With this task, we were able to estimate the contribution of each eye to binocular vision at different interocular contrast ratios. We computed the interocular contrast ratio where two eyes contributed equally in binocular phase combination to quantify interocular suppression. We found that, although the visual acuity of patients’ amblyopic eye was significantly improved by occlusion therapy, the degree of suppression did not significantly change even after 6 months of occlusion therapy. Therefore, our results suggest that the visual benefits provided by occlusion may not be due to reduced suppression from the fellow eye.

## Materials and Methods

### Participants

Ten children with amblyopia (A1–A10) with (*n* = 2) or without (*n* = 8) strabismus participated in our study. All patients were newly diagnosed and had no treatment history before participating in our study. Clinical details of patients before data collection are provided in Table 1. The definition of amblyopia for this study is conducted by PPP [American Academy of Ophthalmology, Preferred Practice Patterns (Christiansen et al., 2018)]: patients had an interocular acuity difference of 2 lines (0.2 logMAR) or more, with an obvious cause (anisometropia, accommodation, strabismus, or deprivation). After 2 months of refractive adaptation (i.e., optical treatment), observers were asked to wear an opaque patch for 4 h each day. Except patient A9 who was followed up to 4 months and patient A10 who was followed only up to 2 months, all patients were followed up to 6 months. Individuals’ visual acuity and interocular suppression were assessed before and after 0.5, 1, 2, 4, and 6 months of occlusion therapy.

**TABLE 1:** Baseline clinical details of the participants.

**Subject**	**Age/Sex**	**Cycloplegic refractive errors (OD/OS)**	**Amblyopia type**	**Squint (OD/OS)**	**Balance point**	**logMAR visual acuity (OD/OS)**
A1 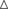	9/M	+3.75	Aniso	Ø	0.37	0.27
		Plano		Ø		–0.03
A2 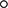	7/M	Plano	Aniso	Ø	0.26	–0.03
		+2.00 + 0.50 × 80		Ø		0.19
A3 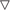	5/F	−1.00 − 1.00 × 180	Aniso	Ø	0.43	0.27
		−6.00 − 2.00 × 180		Ø		0.58
A4 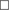	6/M	+1.50	Aniso	Ø	0.29	–0.03
		+5.00		Ø		0.58
A5 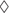	8/M	Plano	Aniso	Ø	0.19	–0.03
		+2.50 + 1.75 × 80		Ø		0.58
A6 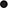	6/M	+3.50	Accom + Stra	Ø	0.16	0.18
		+4.00 + 0.75 × 95		ET5°		0.67
A7 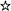	8/F	+4.50	Aniso	Ø	0.33	0.48
		Plano		Ø		–0.03
A8 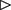	6/F	Plano	Aniso	Ø	0.23	–0.03
		+2.00 + 1.75 × 85		Ø		0.88
A9 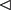	9/M	+4.00	Aniso	Ø	0.13	0.58
		Plano		Ø		–0.12
A10 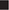	8/F	+3.00 + 0.75 × 90	Accom + Stra	Ø	0.09	–0.03
		+3.50 + 1.00 × 85		ET10°		0.18

*Anis, anisometropia; Stra, strabismus; Accom, accommodation; ET, esotropia.*

### Apparatus

The stimuli for interocular suppression measurement were generated and controlled by a PC computer running Matlab (MathWorks, Natick, MA, United States) with PsychToolBox 3.0.9 extension (Brainard, 1997; Pelli, 1997). The stimuli were presented on a gamma-corrected LG D2342PY 3D LED screen (LG Life Science, South Korea) with a 1920 × 1080 resolution, 8 bits of gray level, and a 60-Hz refresh rate. Subjects viewed the display dichoptically with polarized glasses in a dimly lit room at a viewing distance of 136 cm. The background luminance was 46.2 cd/m^2^ on the screen and 18.8 cd/m^2^ through the polarized glasses.

Participants’ best spectacle-corrected visual acuity was measured monocularly using the Chinese Logarithmic Tumbling E Chart (Mou, 1966) at 5 m. The visual acuity chart has 14 lines; the size of the optotypes changed from 1 to −0.3 logMAR in different lines with a step size of 0.1 log units. Patients were asked to report the orientation (the opening) of the letter “E” one after another and were stopped when they could not respond within 10 s. The amblyopic eye was always examined first during the experiment. Visual acuity was defined as the score associated with 75% correct judgments. This was achieved by measuring participants’ percentage correct at different lines and using linear interpolation to calculate the score associated with 75% correct judgments. Strabismus angle was measured using the prism cover test.

### Design

**FIGURE 1:**
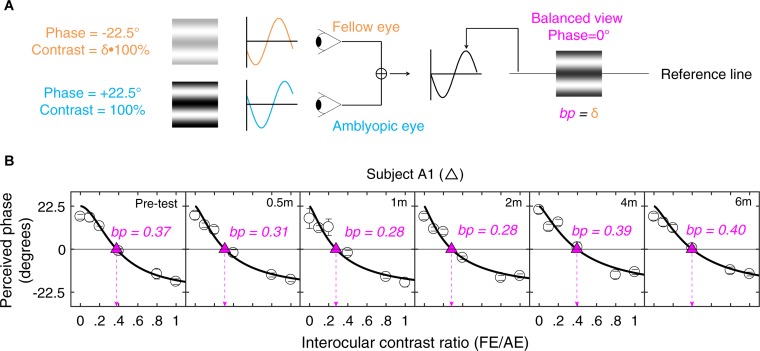
Illustration of the experimental procedure for measuring suppression. **(A)** Our task for quantifying interocular suppression. Two sine-wave gratings with equal and opposite phase shifts are dichoptically presented to the two eyes. The grating seen by the amblyopic eye has a fixed contrast of 100%, while the grating in the fellow eye has proportionally less contrast (ratio less than 1). Interocular suppression index is quantified by the interocular contrast ratio (fellow eye/amblyopic eye) that is needed to achieve a balanced view where the two eyes are equally effective in binocular viewing, i.e., the binocular perceived phase is 0°. **(B)** Example of one subject’s data. Each panel shows a function relating binocular perceived phase to the interocular contrast ratio (PvR). This function was measured at the beginning of study and after short-term occlusion (0.5, 1, and 2 months; all patients participated), after medium-term occlusion (4 months; patients A1–A9 participated), and after long-term occlusion (6 months; patients A1–A8 participated). The data were fitted with the attenuation contrast-gain control model (solid curve) to derive the effective contrast ratio at balance point (where there is equal contribution from each eye to the binocular percept), which is marked as filled triangle and dashed arrow line in each panel. The estimate of the effective contrast ratio at balance point (in short, balance point or “bp”) is provided in each panel. An increase of “bp” toward one indicates less interocular suppression, while a decrease of “bp” toward zero indicates more interocular suppression.

A binocular phase combination paradigm (Ding and Sperling, 2006) was used to quantify the two eye’s contribution to binocular percept (i.e., our measure of interocular suppression). In the test, two horizontal sine-wave gratings with equal and opposite phase shifts of 22.5° (relative to the center of the screen) were dichoptically presented to the two eyes. The contrast was fixed as 100% in the amblyopic eye and varied with a ratio δ (δ = [0, 0.1, 0.2, 0.4, 0.8, 1]) in the fellow eye. These contrast ratios were selected based on our previous studies (Huang et al., 2009; Zhou et al., 2013b) and recent papers in studying the effect of refractive adaptation in children and adults with amblyopia (Wang J. et al., 2018) as well as normative data (Wang Y. et al., 2018). The interocular suppression is quantified by the interocular contrast ratio (fellow eye/amblyopic eye) when the two eyes’ information makes an equal contribution to binocular viewing, i.e., when the perceived phase of the binocularly combined grating is 0° (Figure 1A).

The phase of the binocularly combined grating was measured with an adjustment method for different interocular contrast ratios (δ). To cancel any potential positional bias, two configurations were used in the measurement: (1) the phase shift was +22.5° in the amblyopic eye and −22.5° in the fellow eye; (2) the phase shift was −22.5° in the amblyopic eye and +22.5° in the fellow eye. The perceived phase at each interocular contrast ratio (δ) was quantified by half of the difference between the measured perceived phases in these two configurations. The two configurations at the six interocular contrast ratios were measured eight times using the method of constant stimuli. The perceived phase and its standard error were calculated based on these eight repetitions. There were in total 96 trials (2 configurations × 6 interocular contrast ratios × 8 repetitions) in one measure, which took about 20 min to finish. Voluntary breaks were allowed during the test. Practice trials were provided prior to data collection. The function of perceived phase vs. interocular contrast ratios (PvR function, Figure 1B) was then derived and was fitted by the attenuation gain control model (Huang et al., 2009) to get the effective contrast ratio at balance point (i.e., “bp”) as illustrated in Figure 1. Some patients either found the baseline suppression measurements too difficult or were too variable in their responses; these patients were not enrolled in the study. Only patients who could complete the baseline measurements were enrolled in the study. All the patients who were enrolled in the study subsequently completed the study.

### Stimuli

The stimulus configurations were identical to that previously described (Zhou et al., 2013b): two monocular horizontal sine-wave gratings with different contrast but having equal and opposite phase shifts (relative to the center of the screen) were dichoptically presented in the middle of the two monocular displays. The sine-wave gratings had a period of two cycles, which subtended 2.0° of visual angle (i.e., 1.00 cycle/°). A high-contrast frame (0.11° in width and 6° in length) with four white diagonal lines (0.11° in width and 2.83° in length) was presented surrounding the grating in each eye to help observers maintain fusion. A 1-pixel black reference line was presented horizontally at the two sides of the gratings and observers were asked to move it to indicate the perceived phase after combination.

### Procedure

An alignment task was provided at the beginning of each trial to make sure the two eyes’ images were correctly fused. In the alignment task, a fixation marker was presented in the center of the larger high-contrast frame together with four white diagonal lines. This marker consisted of binocular fixation crosses (100 × 100 pixels^2^) and four monocular dots (20 pixels diameter), two of which were in the first and third quadrants in the left eye and two of which were in the second and fourth quadrants in the right eye. Observers were instructed to move the image in their amblyopic eye using up, down, left, and right arrow keys to align the images from two eyes. After achieving stable fusion, observers were asked to press the “space” key. The corresponding coordinate between two eyes was then used in subsequent measurements. After that, a phase adjustment procedure (Zhou et al., 2014a) was used to measure the perceived phase of the binocularly combined gratings. Observers were asked to adjust the position of the reference line at the side of the grating to indicate the perceived phase of the cyclopean sine-wave grating, defined as the location of the center of the dark stripe of the grating. The reference line was presented with an initial position randomly (−9 to 10 pixels) assigned relative to the center of the frame in each trial. It was moved with a fixed step size of 1 pixel, corresponding to 4° phase angle of the sine-wave grating. During one trial, the gratings, frames, and reference lines were presented continually in the two eyes until subjects finished the phase adjustment. A typical trial lasted for about 10 s.

### Curve Fits

The PvR functions for different dichoptic pairs were fitted with the attenuation gain control model from Huang et al. (2009):

(1) $$\varphi =2\,\tan^{-1}\left[\frac{1-{\left(\delta \,/\,\mathrm{bp}\right)}^{1+\gamma }}{1+{\left(\delta \,/\,\mathrm{bp}\right)}^{1+\gamma }}\cdot \tan\left(\frac{\theta }{2}\right)\right]$$

in which φ is the measured perceived phase when the interocular signal contrast ratio is δ (δ = [0, 0.1, 0.2, 0.4, 0.8, 1.0]); θ is the interocular phase difference (i.e., 45° in our test) and the two free parameters, bp and γ, represent the effective contrast ratio at balance point (i.e., φ = 0°) and the non-linear factor in the binocular combination, respectively.

Curve fits were conducted in Matlab (MathWorks, Natick, MA, United States) using non-linear least squares method to minimized Σ(φ_*theory*_ − φ_*observed*_)^2^.

### Statistical Analysis

All analyses were performed using IBM-SPSS 23.0 (IBM Inc., Armonk, NY, United States). Linear mixed-effects models were applied to explore associations of occlusion duration with visual acuity and interocular suppression. The relationship between changes of visual acuity and interocular suppression was assessed using Pearson correlation analysis.

## Results

### The Effect of Occlusion Therapy on Patients’ Visual Acuity

**FIGURE 2:**
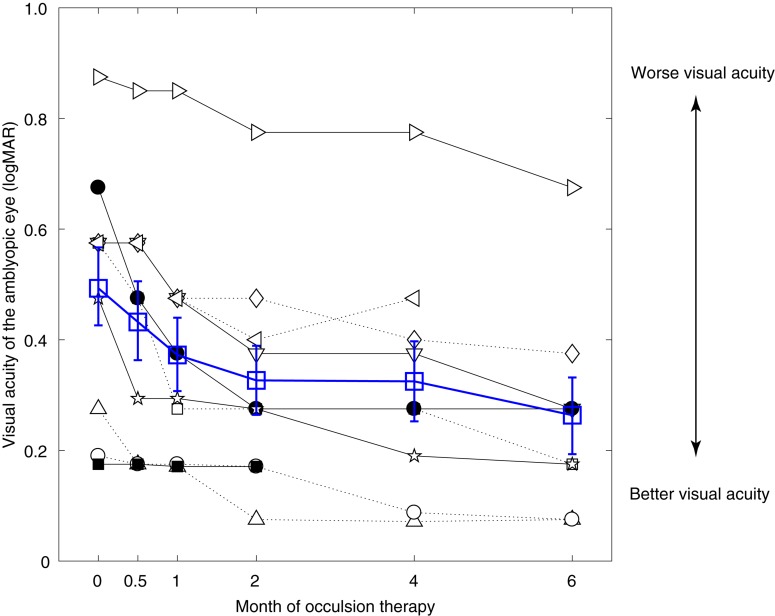
Significant benefits on amblyopic eye’s visual acuity from occlusion therapy. Individuals’ visual acuity of the amblyopic eye as a function of the occlusion duration. Results of different patients were plotted using black symbols, and their average was plotted using blue open squares. Error bars represent SEM.

Figure 2 shows the visual acuity of the amblyopic eye as a function of occlusion durations in the 10 amblyopes (black symbols). The average results are also plotted in Figure 2 using open blue squares. Except patient A10 (■), who showed almost no change of visual acuity, all other patients had clear improvement following the occlusion therapy.

For patients who finished the short-term occlusion therapy (i.e., 2 months; A1–A10), the average visual acuity of the amblyopic eye changed from 0.50 ± 0.22 (mean ± SD) to 0.33 ± 0.20. For patients who finished the medium-term occlusion therapy (i.e., 4 months; A1–A9), the average visual acuity of the amblyopic eye changed from 0.53 ± 0.20 (mean ± SD) to 0.32 ± 0.22. For patients who finished the long-term occlusion therapy (i.e., 6 months; A1–A8), the average visual acuity of the amblyopic eye changed from 0.48 ± 0.19 (mean ± SD) to 0.22 ± 0.10.

A linear mixed-effects model showed that the visual acuity of the amblyopic eye was significantly associated with occlusion durations with the estimated fixed effect being −0.031 (*p* < 0.001). This indicated that the visual acuity of the amblyopic eye improved approximately 0.031 logMAR per month during 6 months of occlusion therapy. On the other hand, the visual acuity of the fellow eye was not significantly changed over time during occlusion therapy (*p* = 0.169). These results suggest that the visual acuity benefits of the amblyopic eye could not be simply accounted for by a practice effect of repeated testing.

### The Effect of Occlusion Therapy on Patients’ Interocular Suppression

**FIGURE 3:**
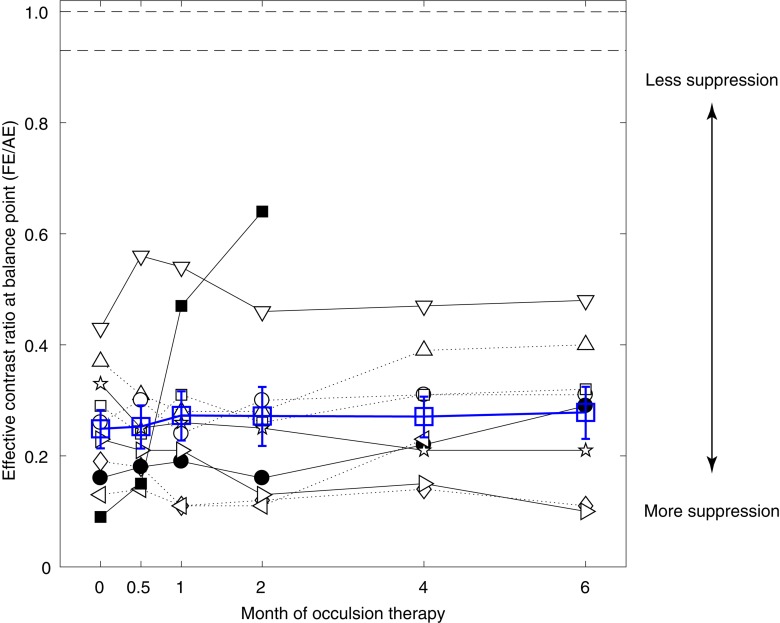
No significant benefits on interocular suppression from occlusion therapy. Individuals’ effective contrast ratios at balance point as a function of the occlusion duration. Results of different patients were plotted using black symbols, and their average was plotted using blue open squares. The two dashed lines at the top indicate the normal range of balance point derived from the literature, which is 0.93 from Wang Y. et al. (2018) (averaged from 144 adults) and 1.0 for ideal observer. Error bars represent SEM.

Patients were well-practiced before beginning the interocular suppression measurements (i.e., the binocular phase combination test). The quality of the data was excellent as the attenuation gain control model from Huang et al. (2009) fitted well to our data. The averaged goodness of fit for the patients were 0.90 ± 0.14 (pre-occlusion treatment; mean ± SD), 0.94 ± 0.04 (after 0.5 months of occlusion treatment), 0.96 ± 0.03 (after 1 month of occlusion treatment), 0.93 ± 0.08 (after 2 months of occlusion treatment), 0.95 ± 0.03 (after 4 months of occlusion treatment), and 0.95 ± 0.05 (after 6 months of occlusion treatment) at different time sessions. Figure 3 shows the interocular suppression (i.e., effective contrast ratio at balance point) as a function of occlusion treatment durations in the 10 amblyopes (black symbols). The average results were also plotted in Figure 3 using open blue squares. Except for patient A10 (■), who showed a dramatic increase of the balance point, all other patients had consistent and similar balance points even after 6 months of occlusion therapy. No patient reached the normal range derived from previous reports in normal adults, i.e., 0.93 (Wang Y. et al., 2018) or the ideal observer’s level (i.e., 1.0).

For patients who finished the short-term occlusion therapy (i.e., 2 months; A1–A10), the average effective contrast ratio at balance point changed from 0.25 ± 0.11 (mean ± SD) to 0.27 ± 0.11. For patients who finished the medium-term occlusion therapy (i.e., 4 months; A1–A9), the average effective contrast ratio at balance point changed from 0.26 ± 0.10 (mean ± SD) to 0.27 ± 0.10. For patients who finished the long-term occlusion therapy (i.e., 6 months; A1–A8), the average effective contrast ratio at balance point changed from 0.28 ± 0.09 (mean ± SD) to 0.28 ± 0.13. A linear mixed-effects model showed that the effective contrast ratio at balance point was not significantly associated with occlusion durations (*p* = 0.309), which indicated no change of interocular suppression during occlusion therapy.

**FIGURE 4:**
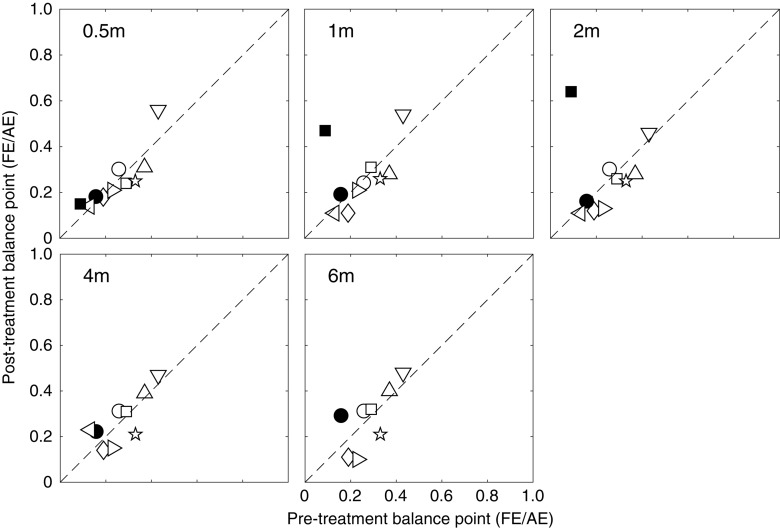
Individuals’ post-treatment balance points as a function of the pre-treatment balance points. Symbols above the dashed line (identity line) indicate less suppression after occlusion, while those below the dashed line indicate more suppression after occlusion.

To better illustrate the change of effective contrast ratio at balance point, post-treatment balance points relative to the pre-treatment balance points are plotted in Figure 4 for each patient at different occlusion stages (i.e., 0.5, 1, 2, 4 and 6 months). It is clear that most patients had a constant and consistent balance point (i.e., close to the identity line) after up to 6 months of occlusion therapy. Based on the effect size (i.e., change of balance point) and the variance in our samples at different occlusion durations, we found that the sample size would have to be at least 545, 2065, and 2742, respectively for 2, 4, and 6 months of occlusion treatment to reach an 80% power and two-tailed significance level at α = 0.05. These results in turn indicate that the change in suppression was not clinically meaningful. This demonstrates that these patients had significant suppression that did not change over the course of the treatment. This can be contrasted with the small but significant change (the average binocular gain was 0.11 in terms of the effective contrast ratio) in suppression that occurs as the result of a similar period of inverse occlusion in a similar age group using the same suppression measuring task (Zhou et al., 2019).

### The Relationship Between Changes of Visual Acuity and Interocular Suppression

**FIGURE 5:**
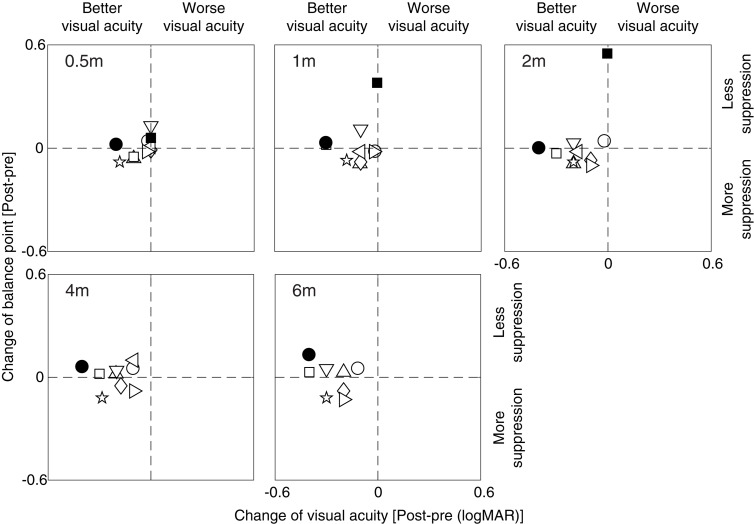
The covariation between balance point change and visual acuity change. Symbols above the horizontal dashed line indicate less suppression after occlusion, while symbols below the horizontal dashed line indicate more suppression after occlusion; symbols at the left side of the vertical dashed line indicate better visual acuity after occlusion, while symbols at the right side of the vertical dashed line indicate worse visual acuity after occlusion.

To better illustrate the relationship between the change of effective contrast ratio at balance point (our measure of suppression) and the change of amblyopic eye’s visual acuity, we plotted post-treatment balance point change relative to the post-treatment visual acuity change in Figure 5 for each patient at different occlusion stages (i.e., 0.5, 1, 2, 4 and 6 months). It is clear that most patients had a constant and consistent balance point (i.e., points close to the horizontal line) and increased visual acuity (i.e., points shift to the left side from the vertical line) after up to 6 months of occlusion therapy. The two visual outcomes were not significantly correlated at any occlusion durations (for all, *p* > 0.10, Pearson correlation analysis). This demonstrates that the change of balance point did not vary with the change of visual acuity.

## Discussion

Occlusion treatment – be it short-term, intermediate-term, or long-term – has been shown to improve visual acuity in about 50% of patients with amblyopia (Holmes et al., 2003, 2005; Repka et al., 2003). Our findings are in line with previous studies regarding the improvement in visual acuity from occlusion therapy. Despite the apparent improvement in visual acuity, the degree of interocular suppression, which was abnormal in all cases, did not change significantly. This was true for all durations of the occlusion treatment regime. However, the findings of Kelly et al. (2016) are in contrast with our results. They show that occlusion treatment for 2 weeks during which the fellow eye was deprived for 2 h per day reduces interocular suppression and facilitates recovery in binocular vision. We do not have any explanations for this change in suppression after a short period of occlusion therapy because our findings show no such change in the short or long terms.

We recruited 10 children with amblyopia (two with strabismus and eight without strabismus) in this study. Although this sample size might appear to be too small to show a robust result, it had been chosen based on previous studies (Moseley et al., 1997; Pediatric Eye Disease Investigator Group Writing Committee Rutstein et al., 2010; Kehrein et al., 2016; Wang et al., 2016; Zhou et al., 2019). For example, the minimum sample size of six patients is adequate to achieve a power greater than 80% in detecting a visual acuity gain of 0.15 ± 0.12 logMAR (data from Wang et al., 2016). Similarly, the minimum sample size of two patients is adequate to achieve a power greater than 80% if the expected binocular gain in terms of the effective contrast ratio is 0.11 ± 0.055 (data from Zhou et al., 2019). The previous studies collectively reinforce the notion that our sample size of 10 patients with amblyopia is adequate to achieve a sufficient power to detect small changes in visual acuity and interocular suppression following occlusion therapy.

However, the visual acuity benefits did not show a significant correlation with reduced suppression in our patients. This finding is consistent with a previous study by Kehrein et al. (2016). They used a qualitative clinical test involving a red filter ladder (i.e., the Sbisa bar test) to quantify interocular suppression. They found no statistically significant change in interocular suppression after 4 months (6 h per day) of occlusion therapy. Their further analysis shows that the change of interocular suppression may be different between the subtypes of amblyopia: interocular suppression slightly declined after 4 months of occlusion treatment in amblyopes without strabismus (7/15), whereas that of amblyopes with strabismus did not (8/15). However, we did not find robust changes of interocular suppression in our anisometropic amblyopes (8/10). Since both studies contain a small number of subjects, whether this is due to the individual variability or a poor test–retest reliability of the quantitative clinical test, even in adults (Piano and Newsham, 2015), remains to be resolved. Furthermore, a luminance-based interocular suppression test (i.e., the Sbisa bar test) in the study of Kehrein et al. (2016) and a more quantitative laboratory, contrast-based interocular suppression test (i.e., the binocular phase combination task) in the current study might reflect different mechanisms that underlie interocular suppression (Chen et al., 2019). Nevertheless, both studies suggest that the visual acuity benefits from occlusion therapy are not correlated with those of reduced interocular suppression. In this respect, it is likely that the benefits from occlusion therapy could be from a different source than those of binocular treatment, which reduces suppressive effects from the fellow eye (Hess et al., 2011; Li et al., 2013; Spiegel et al., 2013). In fact, Mansouri et al. (2008) discovered the treatment effects serendipitously by measuring interocular suppression. They realized over several sessions of measurement that not only the degree of interocular suppression reduced (Mansouri et al., 2008) but also the visual acuity of the amblyopic eye improved (Hess et al., 2010a, b). Subsequent studies have confirmed this relationship (Hess et al., 2011; Li et al., 2013; Spiegel et al., 2013). This is clear in adults from the aforementioned studies but not so in children (although see Kelly et al., 2018), from whom it is more difficult to obtain valid measurements of interocular suppression.

The notion that monocular occlusion therapy and binocular therapy might involve different neural mechanisms is in line with several other notable differences between the effects of these two therapeutic approaches. First, occlusion is only effective in children up to the age of 17, but it is ineffective in adults (Epelbaum et al., 1993). Binocular training has been shown to be effective in adults (Hess et al., 2010a, b, 2011; To et al., 2011; Li et al., 2013; Spiegel et al., 2013) and children (Knox et al., 2012; Li et al., 2014; Birch et al., 2015) with similar effectiveness. Second, a better binocular outcome has been achieved through binocular training than occlusion (Knox et al., 2012). Third, the treatment duration is of the order of 20–40 h for binocular training and over 120 h for occlusion (Stewart et al., 2004; Hess et al., 2010b). Fourth, the recurrence rate is high with occlusion [24–27% (Holmes et al., 2004; Bhola et al., 2006)] and low with binocular training (Birch et al., 2015). Fifth, studies on children using the binocular approach have primarily examined children who failed to improve from occlusion therapy or who reached their best recovery after occlusion therapy (Knox et al., 2012; Li et al., 2014; Birch et al., 2015). Yet, the binocular approach has been shown to achieve additional benefit in visual acuity (Knox et al., 2012; Li et al., 2014; Birch et al., 2015).

Binocular training is based on the idea that reducing the contrast response from the fixing eye will result in less suppression of information in the neural circuits associated with the amblyopic eye (Zhou et al., 2018). This in turn will result in a more balanced interocular inhibition and subsequently enable information from the amblyopic eye to contribute more in binocular vision. Animal models suggest that GABA mediates this form of suppression in the primary visual cortex (Sengpiel et al., 1994, 2006) either via the long-range cortical horizontal fibers connecting large basket cells (Sengpiel et al., 1994, 2006) in superficial cortical layers that connect same and opposite eye domains (Buzas et al., 2001) or via binocular suppression by inhibitory interneurons receiving input from thalamocortical inputs and simple cells, occurring at the thalamo-cortical synapse. If the effects of occlusion do not rely on reduced interocular suppression, what could their neural basis be? One possibility is that it could be due to meta-plasticity (Abraham and Bear, 1996). Meta-plasticity involves homeostatic regulation of synaptic plasticity. In the mouse, monocular deprivation leads to not only synaptic depression of deprived eye synapses but also potentiation of non-deprived eye synapses that is experience-dependent (Frenkel and Bear, 2004; Iny et al., 2006). Thus, by abolishing or severely reducing responses from the fixing eye with an occluder, improved correlation can be obtained between the previously weak synaptic responses and the post-synaptic activity of neurons. The only problem with this explanation is that the predicted response deficit to the occluded eye is rarely observed in the age range where occlusion is used.

If it is the case that these two therapeutic approaches have different sites of action, it raises the question that if they were used together, would their combined effects be mutually constructive or destructive. One suspects that the answer to this question might depend on the outcome measure. If the outcome measure was visual acuity, the two approaches may well constructively combine. However, if the outcome measure was binocular vision (including stereopsis), one might expect a destructive interference; monocular occlusion and binocular combination are, by definition, polar opposite procedures. Two different types of neural recovery in turn suggest two different components to the original neural deficit, one with a binocular basis and another with a monocular basis.

### Relevance to Other Monocular Interventions in Amblyopia

Besides occlusion therapy and binocular training that we have mentioned above, there are several other monocular interventions to improve visual acuity in patients with amblyopia. One example of monocular intervention for treating amblyopia is a non-invasive brain stimulation technique. Thompson et al. (2008) firstly showed that transcranial magnetic stimulation (TMS) could temporarily improve the contrast sensitivity of the adult amblyopic eye. Later, by using daily continuous theta burst stimulation (cTBS) of the visual cortex, Clavagnier et al. (2013) found that the effect of daily cTBS on contrast sensitivity could be accumulated and is long lasting. The findings were also reported by other studies (Ding et al., 2016; Bocci et al., 2018). This is suggested to be a result of altering the balance between excitation and inhibition of targeted brain areas by the non-invasive brain stimulation (Nitsche and Paulus, 2000; Campana et al., 2016). This could well represent a more direct way of redressing the interocular imbalance that is due to suppression; this is yet to be determined. Another example of monocular intervention in amblyopia is monocular visual perceptual learning (PL). It relies on intensively visual training with specially designed visual tasks. There has been evidence that visual PL could improve an amblyopic eye’s Vernier acuity (Levi et al., 1997), visual acuity (Polat et al., 2004), contrast sensitivity (Zhou et al., 2006), and other vision functions (Li and Levi, 2004; Levi, 2005; Huang et al., 2008; Astle et al., 2011). There is evidence that PL can enhance modulation in neuronal tuning in V1 to the trained stimulus and that this consequently facilitates visual functions (Huang et al., 2008; Zhou et al., 2012; Ren et al., 2016; Yu et al., 2016). The role of interocular suppression is less clear for this approach. Two recent studies have tried to answer this question but have come to opposing conclusions: Chen et al. (2016) found that monocular PL improved binocular combination in adult amblyopes. However, by using a similar training protocol, Jia et al. (2018), failed to find any significant effect of monocular PL on binocular phase combination. Considering that the training in these two studies was conducted at a high spatial frequency (cutoff spatial frequency), which was not matched with the spatial frequency of their interocular suppression measurements (0.3 or 1 c/°), it would be necessary for further studies to explore the effect of PL on binocular visual functions using an approach (Kwon et al., 2015; Birch et al., 2016; Wang et al., 2019) that would allow the effect of spatial frequency to be considered for suppression.

Unlike occlusion treatment, both non-invasive brain stimulation and monocular PL are effective in older adults as well and to act on a short time frame, closer to binocular therapies than to occlusion. Furthermore, the effects of daily cTBS and visual PL on amblyopia are long lasting (Li and Levi, 2004; Levi and Li, 2009; Clavagnier et al., 2013), thus most likely exhibiting a smaller remission rate than occlusion. Moreover, Campana et al. (2014) found that PL associated with transcranial random noise stimulation (tRNS) could improve visual acuity and contrast sensitivity substantially. Moret et al. (2018) latterly using a high-frequency transcranial random noise stimulation (hf-tRNS) combined with a short perceptual training showed that hf-tRNS could boost the transfer of PL to untrained visual functions. A strengthening of the low-level responses from the amblyopic eye might result in more balance in the mutual inhibitory interocular circuits and could indirectly result in a reduced suppressive influence. It would also be of interest to investigate the effect of the combination of these monocular interventions on the interocular suppression of amblyopes.

### Limitations of the Present Study

Owing to the spatial resolution limitations of the binocular phase combination task (Wang et al., 2019), our measures of interocular suppression are limited to 1 c/°. In amblyopia, interocular suppression occurs across the entire spatial frequency range (Ding et al., 2013; Kwon et al., 2015; Zhou et al., 2018). Whether the interocular suppression is slightly greater at higher spatial frequencies (Zhou et al., 2018) is controversial because the contrast attenuation caused by the threshold deficit is often not taken into account in such measurements. It is a possibility that occlusion therapy influences interocular suppression in a spatial frequency specific manner and that our measurements of interocular suppression at 1 c/° do not reflect and that binocular therapy and occlusion therapy differ in this respect. Even if this was the case, it would still suggest that their mode of action was different. Another proviso is that we assume that the different techniques that have been developed to measure interocular suppression are all measuring the same suppressive effects. In our study, we use a binocular phase combination task; previous measurements of the effects from binocular therapy have used a global motion approach (Black et al., 2011). A previous study (Zhou et al., 2013b) has shown that the interocular suppression measured with these different tasks while being significantly different is, however, correlated in adults with amblyopia. While we have no reason to doubt that this is not the case in children with amblyopia, we are at present assuming this. Thirdly, to make sure that patients responded to the occlusion therapy, we selected children of 9 years old or younger. Furthermore, all the subjects we studied here had anisometropic amblyopia or mixed amblyopia. We cannot therefore extend our conclusion to patients with a pure strabismic amblyopia.

## Data Availability Statement

The datasets generated for this study are available on request to the corresponding authors.

## Ethics Statement

The studies involving human participants were reviewed and approved by Ethics Committee of the Wenzhou Medical University. Written informed consent to participate in this study was provided by the participants’ legal guardian/next of kin.

## Author Contributions

HC, JZ, and RH conceived the experiments. YC, ZH, and YM performed the experiments. YC, ZH, and JZ analyzed and interpreted the data. YC, JZ, HC, and RH wrote the manuscript. All authors contributed to manuscript revision, and read and approved the submitted version.

## Conflict of Interest

The authors declare that the research was conducted in the absence of any commercial or financial relationships that could be construed as a potential conflict of interest.
